# Comparative modelling of protein structure and its impact on microbial cell factories

**DOI:** 10.1186/1475-2859-4-20

**Published:** 2005-06-30

**Authors:** Nuria B Centeno, Joan Planas-Iglesias, Baldomero Oliva

**Affiliations:** 1 Structural Bioinformatics Laboratory, Research Group on Biomedical Informatics (GRIB), IMIM/UPF. c/ Dr. Aiguader 80. 08003 Barcelona, Spain

## Abstract

Comparative modeling is becoming an increasingly helpful technique in microbial cell factories as the knowledge of the three-dimensional structure of a protein would be an invaluable aid to solve problems on protein production. For this reason, an introduction to comparative modeling is presented, with special emphasis on the basic concepts, opportunities and challenges of protein structure prediction. This review is intended to serve as a guide for the biologist who has no special expertise and who is not involved in the determination of protein structure. Selected applications of comparative modeling in microbial cell factories are outlined, and the role of microbial cell factories in the structural genomics initiative is discussed.

## Review

### Introduction

On the last two decades the development of recombinant DNA techniques has extended the use of microbial organisms to produce target proteins. The enteric bacterium *Escherichia coli *is one of the most extensively used prokaryotic organisms for genetic manipulations and for industrial production of proteins of therapeutic or commercial interest [1,2]. However, bacterial organisms often fail to produce target proteins due to problems related with protein misfolding and protein glycosilation. Yeast and fungal protein expression systems are used for the industrial production of relevant enzymes in such cases [3].

There are two main interests in the industrial production of proteins: i) Redefining the optimal properties of the target protein and ii) Avoiding problems of high-scale production. Knowledge of three-dimensional structure of the proteins may be helpful to redesign a modified protein. Computational prediction methods play an essential role to provide us with structural information of a sequence whose structure has not been experimentally determined. Homology based or comparative modeling [4] is the most detailed and accurate of all current protein structure prediction techniques [5]. Its aim is to build a three-dimensional model for a protein of unknown structure on the basis of sequence similarity to proteins of known structure [6]. Comparative modeling relies on the fact that structure is more conserved than sequence during evolution. Therefore, similar sequences exhibit nearly identical structures, and even distantly related sequences share the same fold [7,8]. Comparative modeling critically depends on the knowledge of three-dimensional structure of homologous proteins. The progress of structural genomics initiatives [9] allow to model a large amount of protein sequences. Besides, the number of unique structural folds that proteins adopt is limited {Zhang, 1997; #81; Liu, 2004 #48}. Consequently, it is likely that at least one example of most structural folds will be known, making comparative modeling applicable to most protein sequences. In term, an essential step of structural genomics is production of target proteins. Microbial cell factories play a key role in this context.

This review is intended to give a primer addressed to scientists of disciplines related to microbial cell factories who has no expertise in comparative modeling. Our goal is to provide the seeding background to understand concepts, opportunities and challenges of comparative modeling. We will describe each step in the comparative modeling process, discuss the most common errors and how to solve them, as well as outlining the applications of comparative modeling in the field of microbial cell factories.

We will emphasize the simplest and most reliable methodologies to follow up along with their range of application with a reduced number of useful programs and web servers. Many other authors have also written excellent reviews on the comparative modeling field [6,10-14].

### Steps in comparative modeling

**Figure 1:**
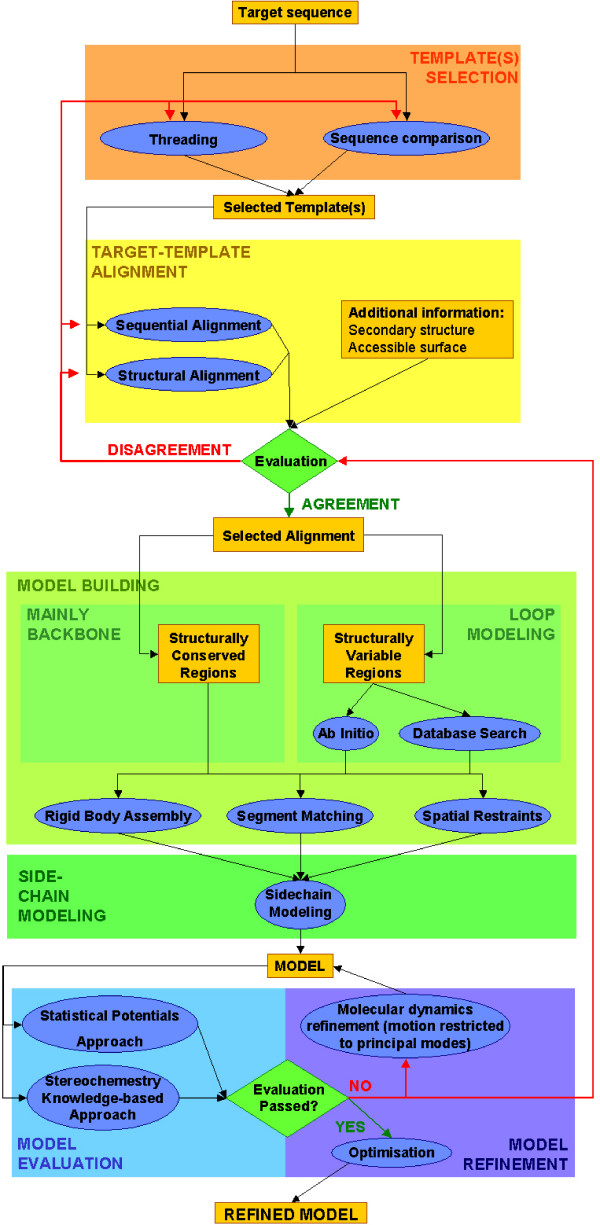
Flowchart of methods used for comparative modeling. Scheme of the methods used for comparative modeling, comprising template(s) selection, template-target alignment, model (backbone and loops) building, sidechain modeling, model evaluation, and model refinement steps. Programs and servers referring to these steps are listed in table 1.

All current comparative modeling methods consist of four sequential steps: template selection, target-template alignment, model building and model evaluation. Essentially, this is an iterative procedure until a satisfactory model is obtained (Figure 1). In this process a variety of programs and web servers can be used (Table 1). Additionally, protein modeling meta-servers are emerging. They automatically implement the full process in a multi-step protocol, using simultaneously different methods [15].

**Table 1:** Useful servers and programs for protein comparive modeling.

**PROGRAM **	**Server/Web adress **	**Reference **
**Template Selection **		
PSI-BLAST		[20]
HMMER (HMM search)		[35]
TOPITS		[17]
FUGUE		[26]
Threader		[27]
3D-PSSM		[28]
PFAM		[25]
PHYLIP		[31]
**Target-Template alignment **		
CLUSTALW		[34]
HMMER (HMM align)		[35]
STAMP		[36]
CE		[37]
DSSP		[38]
**Model Building **		
COMPOSER		[39]
SwissModel		[41]
3D-JIGSAW		[44]
MODELLER		[46]
**Loop Modeling **		
MODLOOP		[50]
ARCHDB		[51]
Sloop		[52]
**Sidechain Modeling **		
WHAT IF		[55]
SCWRL		[56]
Evaluation of the model		
PROCHECK		[67]
PROSA II		[70]
Biotech		
Refinement		
GROMOS		[74]
CHARMM		[75]
AMBER		[76]

#### Template selection

The starting point in comparative modelling is to identify protein structures related to the target sequence and then to select those that will be used as templates. Such templates may be found by sequence comparison methods or by sequence-structure methods also known as threading methods. Sequence comparison methods can be safely used above a certain threshold in terms of sequence identity (i.e. percentage of identical paired residues in an alignment). It has been shown that above that threshold -which is strongly dependent on sequence length, sequence homology implies structural identity [16]. Even though, below that threshold structural likeness is still possible. Some protein pairs sharing very little sequence similarity may have become similar by convergent or divergent evolution. The alignment of these proteins pairs, which define the so called "midnight zone" in sequence alignment [17], is usually addressed with threading methods. Finally, there is a range in terms of sequence identity amid the safe zone and the "midnight zone" in which the relationship between structural homology and the phylogeny is unclear: the "twilight zone" [18]. Within this range, which is usually defined between 20 and 35% sequence identity [19], additional caution must be taken on the sequence alignment.

PSI-BLAST [20], an iterative sequence comparison method, is probably the most widely used program to detect remote similarities. In more difficult scenarios, where sequence homology is not so evident, templates can be found by searching in sequence space using intermediate sequence search (ISS) methods [21-23].

Sequence comparisons can also be made through Hidden Markov Models (HMMs) [24] as implemented, for instance, in HMMER. HMMs profiles of protein domain families are available in Pfam database. These profiles can be used to automatically identify protein domain(s) within the target even if it shares weak sequence similarity with templates [25].

Threading methods have been developed to find more distant relationships. For this reason, they are the most promising choice in the absence of homologues to the target sequence. Threading methods involve performing sensitive sequence searches and characterizing sequence compatibility with the structural environments of putative templates. Features analysed by this kind of methods include secondary structure and solvent accessibility predictions as well as functional annotation. Most used methods of this kind are TOPITS [17], FUGUE [26], Threader [27] and 3D-PSSM [28]. Recent examples of the combined use of these servers and further modeling [29] prove its use. Other methods are being developed based on the analysis of protein-protein interactions to search for remote similarities [30].

Once a list of related proteins with known structure has been obtained, it is necessary to select those templates that are appropriate for the given modelling problem. The feasibility of a template can be assessed by means of its expectation value, E-value [20], which is one of the parameters in the searches outputs. As a general rule, the lower the E-value, the better the template is.

Besides, several other factors should be considered when selecting a template:

1) Quality of the experimental template structure. Because errors in templates will be passed onto the models, the better templates are the most accurate structures available. Accuracy of the templates can be assessed by the resolution and the R-factor for a crystallographic structure, or by the number of distance restraints per residue in the case of NMR structure.

2) Environment likeness. Experimental factors of interest for the target (i.e. the presence of a ligand in the structure, pH, and solvent features ...) should be found as similar as possible in the chosen templates.

3) Phylogenetic similarity. It is helpful to build a multiple alignment and a phylogenetic tree [31] of the target and templates, in order to select templates from the subfamily that is closest to the target sequence. The phylogenetic tree can be constructed by means of PHYLIP set of programs [31].

Depending on the purpose of the model, some of the factors listed above will be more important than others. For instance, resolution of the template is probably the most important factor if the reason for building the model is to design mutants of a binding site, since an accurate geometrical description is needed.

It is important to emphasize that it is not mandatory to select only one template. Actually, methods using multiple templates seems to perform better than those based on a single template [14,32], especially if the main modes extracted from them are taken into account [33]. Finally, it is noteworthy to be aware that, implicitly, choosing templates means the recognition of the target's overall fold.

#### Target-template alignment

Once templates have been selected, an optimal alignment between the target sequence and templates is needed to further construct a three dimensional model of the target.

From easiest to more complex, some strategies for aligning target and templates are:

1) Obtaining a multiple alignment of the templates and the target using CLUSTALW [34].

2) Aligning the query sequence to a HMM profile of the templates family built from a Pfam alignment [25] using HMMER [35].

3) Aligning the query sequence to a HMM profile of the templates built from a structural alignment using HMMER [35]. Structural alignments required for this strategy can be obtained using STAMP [36] or CE [37]; an automatic web server is available for the later one.

In our experience this third strategy, in which not only the sequence similarity but also the structural information inherent in the templates guide the alignment, make it more trustworthy.

The obtained alignments must be critically evaluated in terms of the number, length, and position of the gaps opened. Some of them can be manually refined, taking into account the secondary structure of the templates and their accessible surface, both of them calculated with DSSP program [38], in order to avoid gaps which are opened within secondary structural elements. This will be important if the alignment strategy is based only on sequence similarity. In any case, at this step, if necessary, the selection of templates may be revisited, either to search a new template to overcome a gap in a particular region or to remove redundant or inadequate templates.

#### Model building

Comparative model building generates an all-atom model of the sequence based on its alignment to one or more templates. It includes either sequential or simultaneous modelling of the core of the protein, loops, and side-chains.

The original comparative approach, which is still widely used, is modelling by rigid-body assembly [4]. This method constructs the model from a few regions which are obtained from dissecting related structures. In order to assemble the dissected parts, a framework is calculated by averaging of Cα atoms of structurally conserved regions in template structures. Structurally variable regions are modeled by choosing from a database of all known proteins those regions that better fit the anchor conserved regions. COMPOSER [39] is one of the programs that use this methodology in a semiautomated procedure. SwissModel is a commonly used automated web server also based in this approach [40-42].

Modeling by segment matching is another approach which relies on the approximate positions of conserved atoms in templates [27,43]. This is accomplished by breaking the target into a set of short segments, and searching in a database for matching fragments which are fitted onto an initial framework of the target structure. Database searching is based on sequence similarity, conformational similarity and compatibility with the target structure. 3D-JIGSAW is one of the successful programs that uses this approach [44]

Another approach is modeling by satisfaction of the spatial restrains obtained from the alignment [45]. Probably the most used program based on this approach is MODELLER [46]. First, this automated procedure derives many distance and dihedral angle restraints on the target sequence from its alignment with template three-dimensional structures. Next, this homology-derived restraints and energy terms ensuring proper stereochemistry are combined into a function. Finally, the model is obtained by optimizing this function in such a way that the model violates the input restraints as little as possible. Several slightly different models in agreement with the restraints can be calculated.

Any of the three methods above described produce models of similar accuracy if they are optimally applied. In the difficult cases, modeling by satisfaction of spatial restraints is perhaps the most accurate technique, since it can use many different types of information about the target sequence. In this way, available experimental data can be added as new restraints, making the model more reliable.

#### Loop modeling

Along with alignment, loop modeling is probably the most difficult step in comparative modelling process. Errors in loops are the dominant problem in comparative modelling when target and template share above 35% sequence identity. This is a very active area of research and it is not practical to consider all available methods (details of some of them can be found in [47-49]). In this review we will present the state-of-the art of methods that can be easily used. Furthermore, it must be pointed out that although existing methods can provide reasonably accurate models of short loop regions; modeling of long loops is still an unsolved problem [11]. Loop modeling methods can be classified in two approaches: ab initio methods and database searching or knowledge-based methods.

Ab initio loop prediction is based on a conformational search guided by a scoring or energy function -the later describing the physico-chemical properties of a protein and its environment. There are many such methods, making use of different protein representations, energy functions and optimisation procedures [11]. Among them, there is an option to use implemented in MODELLER or in a web server MODLOOP [50].

The database approach to loop prediction begins by finding segments of main chain that fit the two stems of a loop. The stems are defined as the main chain atoms that precede and follow the loop but are not part of it. The search is performed through a database of many known protein structures, not only homologues of the modeled protein. Usually, many different hits are obtained and possibly sorted according different criteria (geometric or sequence similarity). The selected segments are then superposed and annealed onto the stem regions. These initial crude models are often refined by optimisation of some energy function. Databases searching approach is more accurate and efficient if it is precedent by an structural classification of the loops present in the database. Web-servers based on structural classification [51,52] are available (see table 1). When database searching is used, it must be keep on mind that the bigger the length of the loop, the lesser the number of putative solutions that will be in the database. At the present, this fact makes this approach specially useful for loops up to 7 residues long [53].

Finally, it must be remarked that prediction of a loop conformation is hindered by two main factors: i) the exponential increase of number of possible conformations as the length of the loop grows, and ii) the conformation of a loop is influenced by the core stem regions that span the loop as well as by the structure of rest of the protein that encircles the loop. These two factors make loop modeling one of the most difficult tasks of the comparative modeling process.

#### Sidechain modeling

Similarly to what happens in loop modeling, sidechain conformation can be predicted from knowledge-based approaches or taking into account steric or energetic considerations [54].

Knowledge-based approaches, which are the most widely used, employ libraries of common rotamers extracted from high resolution X-ray structures. Rotamers are tried successively and scored with a variety of energy functions [13]. This approach is implemented in most automatic homology modeling procedures. Among the available software to do so it is worth mentioning: the CORALL module of WHATIF [55] and the SCWRL program [56]. Several works have probed the biological relevance of side-chain modeling, as they may imply behavioural changes in protein-protein interactions and dimerization [57-59].

### Errors in comparative models

Structural models obtained by homology will have regions that resemble the true structure and regions that do not. That is, all models contain a certain amount of errors, which are more frequent as sequence identity decreases. Any stage of the comparative modeling process has its own source of errors; accordingly, they can be divided in five categories [6]:

1) Incorrect templates. This is a problem when templates share less than 25% sequence identity with the target.

2) Missalignments errors. Accuracy of the alignments is still the key limitation on the quality and usefulness of the models, being the optimal placement of gaps its limiting factor [60]. If the target and the templates have over 40% sequence identity, the alignment is almost always correct. As percentage of identity decreases, regions of local low sequence similarity appear, and alignment errors are more feasible to occur. Alignment errors increase rapidly below 30% sequence identity and become the major source of errors in this kind of models [11,14]. Target-template alignment is probably the most crucial step in comparative modelling, since any errors at this step are usually impossible to correct later [47]. Therefore, it is indeed important to devote efforts to attain the most precise alignment.

3) Structural distortions in correctly aligned regions. As sequence identity decreases, it is possible that a segment correctly aligned adopts different local structure than the target, without disruption of the overall fold. It is convenient to use multiple templates whenever they are available to overcome this problem [61].

4) Errors in regions without a template. Insertions are the most challenging regions to model, because there is not a equivalent region in the template. The complexity of the problem increases with the length of the segment. Database searching [62] or energy-based methods [63] can be applied to predict the conformation of the insertion. If there are alignment errors at stem residues or at the other environment residues, insertion modeling is not likely to result in an accurate model [49]. Therefore, the most accurate environment surrounding the insertion, the better results are obtained.

5) Errors in sidechain packing. As sequence identity decreases below 30%, there is a rapid decrease in the conservation of sidechain packing. That is, rotamers of identical residues are not conserved because the overall surroundings are changed. In addition, it must be pointed out that the correct prediction of sidechain conformation is hampered by the coupling between mainchain and sidechains and by the continuous nature of the distribution of dihedral angles [54]. This kind of error can be critical if affecting residues implicated in protein function. As we will see later, a refinement of the structure by energy minimization or molecular dynamics can sometimes surmount this problem [64].

Summarising, consequences of the errors are more serious if they are made in the initial steps of the comparative modeling process: if the selection of the template is wrong, the model based on it will be wrong; if the alignment is incorrect, local features of the model will be incorrect. Remaining errors are mainly due to incorrect description of the environment of a particular region of the structure.

### Evaluation of the models

The quality of the obtained model establish the limits of the information than can be safely extracted from it. Although all structural models obtained by enclose mistakes, they become less of a problem when it is possible to detect them. Once an error is identified, it is possible to discriminate whether it affects key structural or functional regions. Accordingly, strategies to surmount errors should be taken in consideration. Therefore, an essential step in the comparative modeling process is the detection of wrongly modelled regions.

There are two different approaches to estimate errors in a structure: 1) checking the consistency of the model with experimental data of the target protein, and 2) evaluating stereochemistry and other spatial features of the model by means of methods based on statistics derived from experimentally determined protein structures.

On the first approach experimental data is used to certainly determine if particular regions of the protein are correctly modeled. Biochemical data of the most important residues regarding protein overall structure and function can be used to validate the model [65,66]. That is, they should be in close proximity in 3D space and in the correct orientation to perform their role. A consistent modeling of such residues does not ensure a good prediction; conversely, inconsistency is a important reason for concern.

One essential requisite for a model is to have a good stereochemistry. Programs used to check the stereochemistry are based in the analysis of datasets of experimentally determined protein structures. With this respect, the most widely used program is PROCHECK [67], which provide an assessment of the overall quality of the structure and highlight regions that may need further investigation.

Besides stereochemistry, there are other spatial features in the proteins, that could be used as indicators of errors in the models: packing, creation of a hydrophobic core, residue and atomic solvent accessibilities, spatial distribution of charged groups, distribution of atom-atom distances and main-chain hydrogen bonding structures [47]. This kind of information is exploited in another group of programs based on the use of energetic profiles introduced by statistical criteria [68,69]. PROSAII [70] is probably the most widely used program of this category. Although there is a concern about the theoretical validity of the energy profiles for detecting local error in models [6], this approach have been successfully applied [71,72].

It is important to note here that it is highly recommended to analyse the experimentally determined structure of templates with PROCHECK and PROSA II programs. This should allow to discriminate between errors coming from the model and errors already present in the templates.

As a final step, energy minimization and/or molecular dynamics simulations [73] of the model can be done to minimize errors detected with PROCHECK and PROSA II. The most common used programs for this purpose are GROMOS [74], CHARMM [75] and AMBER [76], which explore and evaluate the multiple possible conformations of the protein.

Performing this step is still a controversial issue [77], because the description of the physico-chemical properties of the protein and its environment is not accurate enough [11]. Even though, new evidences are suggesting that long molecular dynamics simulations with explicit solvent could overcome errors in comparative modeling [64]. Over more, strategies focusing on the appropriate sampling of biologically relevant conformations of the protein have been proved to be useful refining the model. This can be achieved by restraining the movement of specific aminoacids [78] or to particular directions in the space [33].

### Comparative modeling applications in the field of microbial cell factories

#### On structure-function relationships

Besides other general applications of protein comparative modeling [6], there are two of them which can be of particular interest in microbial cell factories:

1) Proposing residues for site-directed mutagenesis experiments in target proteins to assess its biological function. There are many examples of how comparative modeling has been used to propose mutants, dealing with different structural features of the protein, such as electrostatic charge and surface shape [79], loop flexibility and residue accessibility [80], the protein binding or enzyme active site [81] or an enzyme alosteric site [82], among others. It is not prudent to apply comparative modeling for this purpose if the target and templates do not share at least around 30% sequence identity, since the required degree of resolution of the model will be not enough to describe the affected structural features on the target protein [6].

2) Detecting an functional important regions of a protein. The knowledge achieved in the process must allow to design proteins with altered or improved functionality. The location of a binding site can be identified by localizing clusters of charged residues [83,84] or using data of deleterious mutations [82] Biological important regions tend to be predicted better than other parts of the model [14], because amino acids in the active and binding sites are often more conserved than other structural features in a protein [85]. In addition, activity is mostly based on the physicochemical properties of residues and its spatial orientation [86]. Consequently, the degree of sequence similarity shared by the target and the templates is less restrictive for this particular application, and thus homology modeling can be applied in a wide range of scenarios, including when sequence similarity drops below 30%.

#### On solving protein production related problems

There are other topics in protein production processes by means of cell factories in which structural-related features play a major role. One example is protein aggregation leading to bacterial inclusion bodies, which constitute a major bottleneck in protein production [87]. Recently, it has been shown that aggregation depends on specific interactions between solvent-exposed hydrophobic stretches which adopt the form of β-sheet structures [88]. This structural knowledge provides some insight on how to solve this problem: such interactions should be specifically disrupted to avoid aggregation of β-sheets. However, full understanding of this phenomena requires also comprehending the structural details on how two or more proteins interacts. This constitutes a challenging problem known as protein-protein docking prediction [89,90]. Recent works suggest that comparative modeling can be still helpful in combination with other experimental techniques to adress this problem [91,92].

### On the meeting point of comparative modeling and cell microbial factories: structural genomics

A major necessity of medium- to high-scale protein production has recently arose with the development of initiatives on structural genomics [93,94]. These initiatives, which pursue to elucidate the tree-dimensional structures of all proteins [95,96], demand optimized and further robotized protein expression systems [87]. This aim will be achieved by a focused, large-scale determination of protein structures by X-ray crystallography and NMR spectroscopy, combined efficiently with accurate protein structure modeling techniques [6].

Structural genomics, as a first step, involves ensuring that each family of proteins is represented by a known structure, avoiding unworthy efforts that will result in redundant structural information. It must be pointed out that, nowadays, there are still families of proteins which must be excluded for this kind of large-scale studies. These problematic cases include integral membrane proteins, highly disulfide-bridge proteins and large complexes [87]. All projects employ exhaustively computational methods for target selection and family exclusion [97]. For the rest of proteins, three-dimensional models can be inferred from the previously resolved family representatives. As a result, a huge amount of structural data will be available, which in turn can serve as starting point for a rational protein production design.

A complete success of the structural genomics initiative critically depends on the advances in protein production technologies. This includes new approaches in expression of targets that show challenges on protein folding [87] and also in the development of automated or semi-automated methods, robust and inexpensive for protein purification [96].

## Conclusion

We have attempted to establish the capabilities and limitations of current methods of comparative modeling, as well as a general strategy to follow up in a practical case, that hopefully could serve as a guide for biologist in this field. This methods are becoming important as tools for scientists working in microbial cell factories. We have shown in this review few examples where the use of comparative modeling have been used in this area.

Comparative modeling can be safely used when target and templates share at least 30% sequence identity. Below this threshold, modeling becomes a difficult task even for experts. In any case, models must be critically evaluated to be sure that they are correct enough, devoting most of efforts to the region involved in function.

Many challenging aspects of comparative modeling are active areas of research. The state-of-the-art of the protein structure prediction strategies and methodologies is tested every two years in the CASP (Critically Assessment of techniques for protein Structure Prediction) meeting. A carefully reading of the proceedings of the meeting is probably the best way to update the progress made by the field. See supplement 6 of volume 53 of Proteins for the last report available [98].

As a final advice, it is a good policy make use of different strategies to build the model and compare them. This is always pertinent but specially as sequence identity decreases. Consistency between different models does not ensure a good prediction; however, inconsistency is a meaningful cause of concern.

With the help of structural genomics, the structure of at least one member of the most globular folds will be determined in the next years, making comparative modeling more easy. However, this is not already true for membrane proteins, which constitute a more difficult scenario [99], and more improvements in both structure determination and modeling techniques are needed.

Finally, we do believe that comparative modeling should play key role in the microbial cell factories. It will help biologists to choose which are the most interesting mutant proteins to produce, to design new proteins with a desired function, or to modify a protein to avoid production-related problems.

## List of abbreviations

Target: protein to be modelled. Templates: set of proteins, homologous to the target, for which three-dimensional structure is known. Model: inferred three-dimensional structure of the target. NMR: Nuclear Magnetic Resonance. HMM: Hidden Markov Model. Structural alignment: sequence alignment based on structural similarities. Cα : Alpha-carbon; carbon atom joining the carboxyl group and the amino group in an amino acid. Restraint: as referred to in this paper, a restraint is a reduction of the conformational space of a protein on account of a prior knowledge. Main chain: sequence of atoms within a protein formed by the carboxyl group, the alpha-carbon and the amino group of each of its amino acids. Side-Chain: atoms of an amino acid not belonging to the main chain. Stem: structured boundary of a loop. Rotamer: a particular conformation of the side-chain of an amino acid regarding the position of its main chain

## Authors' contributions

NBC reviewed the comparative modeling sections and updated its methods. JP reviewed the protein expression systems and the applications of comparative modeling to the microbial cell factories field. BO coordinated the design and redaction of the manuscript. All authors read and approved the final manuscript.
